# High Prevalence of Influenza D Virus Infection in Swine, Northern Ireland

**DOI:** 10.3201/eid3105.241948

**Published:** 2025-05

**Authors:** Paula Lagan, Ken Lemon

**Affiliations:** Veterinary Sciences Division, Agri-Food and Biosciences Institute, Belfast, Northern Ireland

**Keywords:** influenza D virus, viruses, influenza, respiratory infections, swine disease, swine adaptation, Northern Ireland

## Abstract

We detected influenza D virus in multiple swine herds in Northern Ireland. Whole-genome sequencing showed several circulating genotypes and novel mutations in the receptor-binding site and esterase domains of the hemagglutinin-esterase fusion protein. Transmission routes of influenza D virus to swine remain to be clarified but could be direct or indirect.

Cattle are the main reservoir of influenza D virus (IDV) worldwide, although the virus is occasionally detected in other species, including swine (*1*). In Europe, surveillance for IDV in swine has either failed to detect the virus by molecular methods or detected the virus at low prevalence, at <5.6% at the herd level (*2*). Cattle in Northern Ireland have previously tested positive for IDV (*3*). Here, we describe detection of IDV in multiple swine herds from Northern Ireland and report on the genetic characterization of the swine-origin IDV strains.

## The Study

Veterinarians visited 17 swine breeding units, composed of a mixture of farrow-to-wean and farrow-to-finish operations, during January–May 2023. The farms were involved in an ongoing regional control program for porcine reproductive and respiratory syndrome virus (PRRSV) that targeted 10- to 12-week-old pigs according to the PRRSV testing algorithm (https://www.cafre.ac.uk/wp-content/uploads/2023/10/Area-Regional-Control-Final-Report.pdf) for growing pigs. In addition to serum samples for PRRSV testing, 30 nasal swab samples per unit were obtained from the same cohort of growing pigs and tested for the presence of influenza A virus (IAV) and IDV by real-time reverse transcription PCR (rRT-PCR), as previously described (*4*,*5*). Serum samples were sent to a commercial diagnostics company (aCare Lab, https://acarelab.com) for determination of PRRSV status by rRT-PCR and open reading frame 5 gene sequencing. Farms were sampled on 1 occasion, except for farm 2170, which was originally sampled in February and resampled in May at the request of the farmer because of a deteriorating clinical situation. 

**Figure 1:**
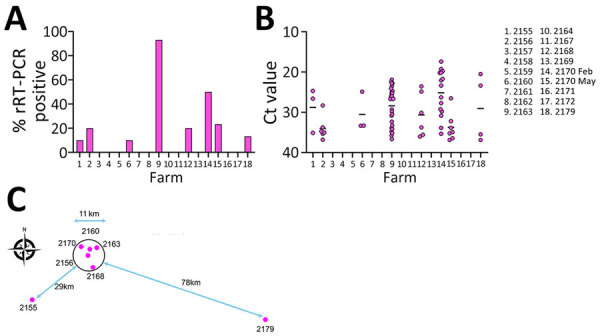
Molecular detection of influenza D virus (IDV) infection in swine, Northern Ireland. A) Percentage of nasal swab samples (n = 30) collected from 10- to 12-week-old growing pigs testing positive for IDV RNA by rRT-PCR. B) Plot of Ct values for IDV-positive rRT-PCR nasal swab samples per farm. Horizonal lines indicate mean Ct values. C) Locations and proximity of IDV-positive swine units. Ct, cycle threshold; rRT-PCR, real-time reverse transcription PCR.

Samples from 7 units (41.2% of herds) tested positive for IDV and had sample positivity of 10%–93% (Figure 1, panel A). Farm 2170 tested positive for IDV at both timepoints. Associated rRT-PCR cycle thresholds ranged from 17.4 to 36.9 (median 28.0) (Figure 1, panel B). The highest rates of sample positivity were 93% for farm 2163 and 50% for farm 2170; the lowest average cycle thresholds were 28.4 for farm 2163 and 25.1 for farm 2170. Four of 7 IDV-positive farms were also positive for IAV, and 3 of 7 were positive for a field strain of PRRSV (Table). Although clinical signs such as coughing and fever were not recorded at the time of sampling, we retrospectively asked producers whose farms tested positive for IDV about the clinical situation on the farm. Three farms (2160, 2163, 2170) reported increased death and slow growth, 3 (2155, 2156, 2166) reported no overt clinical signs, and no response was received from 1 farm (2179) (Table). A follow-up investigation with the attending veterinarians revealed that 5 of the positive farms (2160, 2163, 2168, 2170, 2179) also kept cattle, whereas 2 farms (2155, 2156) were swine-only farms. Cattle grazed in pastures adjacent to the IDV-positive swine-only units, and >1 worker on each swine unit also worked on the nearby cattle farms. Five of the IDV-positive units were located in a swine-dense region, within a 5.5-km radius (2160, 2163, 2168, 2156, 2170), whereas 2 farms were outside that region: 29 km to the southwest (farm 2155) and 78 km to the southeast (farm 2179) (Figure 1, panel C; Appendix).

**Table:** Coinfection status, clinical signs, and links to cattle of sampled farms showing high prevalence of influenza D virus infection in swine, Northern Ireland*

Farm no.	Farm type	IAV status (type)	IDV status	PRRSV status (strain)	Clinical signs	Multispecies farm
2155	Farrow-to-finish	Negative	Positive	Negative	None	Cattle graze beside unit, and 1 worker works on cattle farm
2156	Farrow-to-finish	Positive (untyped)	Positive	Negative	None	Cattle graze beside unit, and 1 worker works on cattle farm
2157	Farrow-to-finish	Inconclusive	Negative	Negative	Unknown	No cattle
2158	Farrow-to-wean	Negative	Negative	Negative	Unknown	Extended family keeps cattle
2159	Farrow-to-finish	Negative	Negative	Inconclusive	Unknown	Dairy cattle on farm
2160	Farrow-to-finish	Positive (H1N2)	Positive	Positive (field strain)	Death and slow growth	Unspecified cattle on farm
2161	Farrow-to-finish	Negative	Negative	Positive (field strain)	Unknown	Extended family keeps cattle
2162	Farrow-to-finish	Positive (pH1N1)	Negative	Negative	Unknown	Dairy cattle on farm
2163	Farrow-to-wean	Negative	Positive	Positive (field strain)	Death and slow growth	Unspecified cattle on farm
2164	Farrow-to-finish	Inconclusive	Negative	Positive (field strain)	Unknown	No cattle
2167	Farrow-to-finish	Positive (untyped)	Negative	Positive (field strain)	Unknown	Beef cattle on farm
2168	Farrow-to-finish	Negative	Positive	Negative	None	Unspecified cattle on farm
2169	Farrow-to-finish	Negative	Negative	Negative	Unknown	Beef cattle on farm
2170	Farrow-to-wean	Positive (H1N2)	Positive	Positive (field strain)	Death and slow growth	Unspecified cattle on farm
2171	Farrow-to-finish	Negative	Negative	Negative	Unknown	Dairy cattle on farm
2172	Farrow-to-finish	Negative	Negative	Negative	Unknown	Beef cattle on farm
2179	Farrow-to-finish	Positive (untyped)	Positive	Negative	Unknown	Unspecified cattle on farm
*IAV, influenza A virus; IDV, influenza D virus; PRRSV, porcine reproductive and respiratory syndrome virus.						

**Figure 2:**
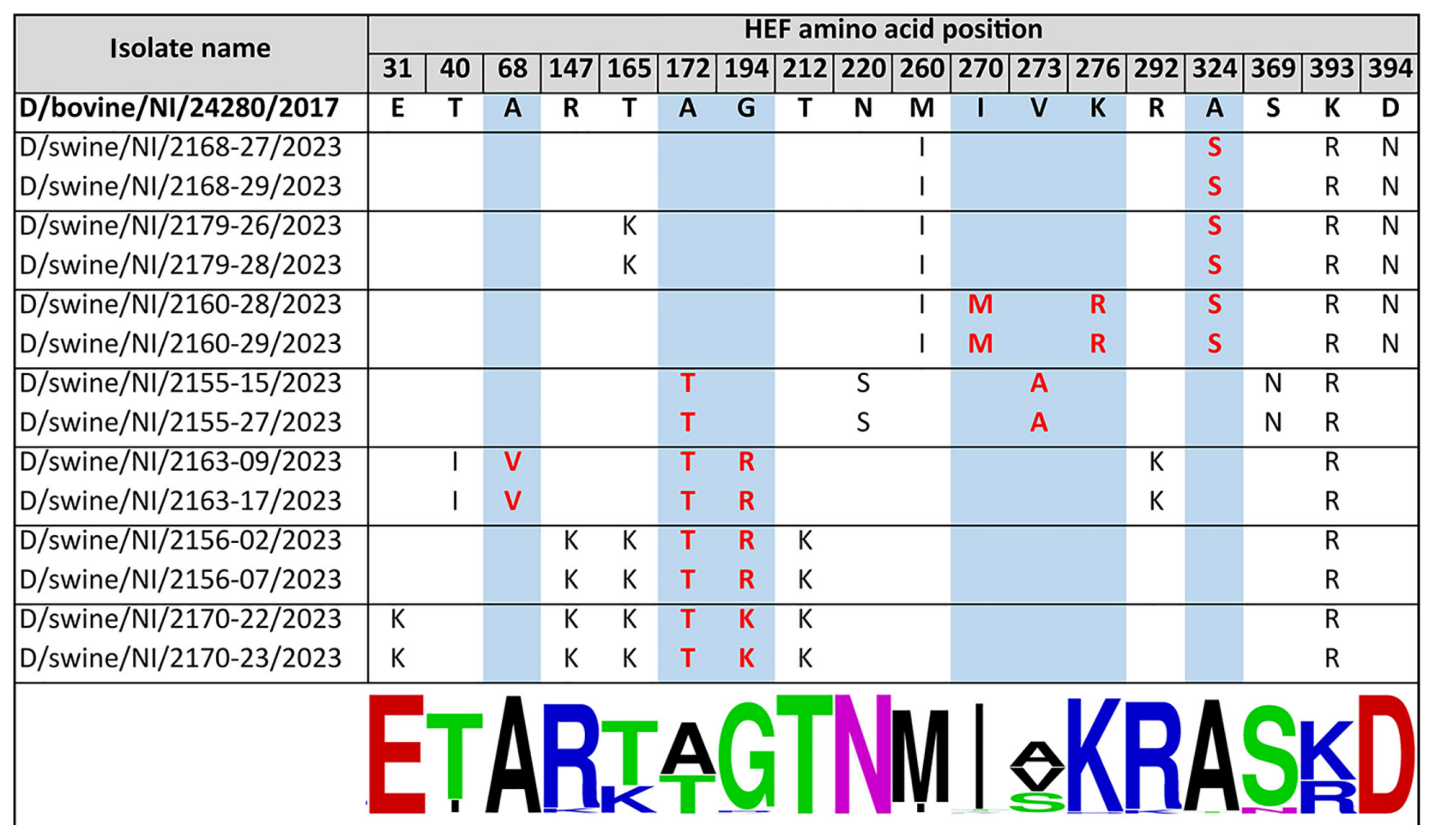
Amino acid substitutions in swine-origin influenza D virus HEF protein relative to bovine-origin influenza D virus, Northern Ireland. Changes relative to the bovine-origin sequence are displayed. Amino acid numbering is according to Song et al. (*6*). Substitutions occurring at the receptor-binding site and esterase are highlighted with red text on a blue background. Sequence conservation at each position is indicated by logo generated at WebLogo (https://weblogo.berkeley.edu/logo.cgi) (bottom) on the basis of alignment of 169 influenza D virus sequences. HEF, hemagglutinin-esterase fusion; NI, Northern Ireland.

We compared hemagglutinin-esterase fusion (HEF) amino acid sequences from swine-origin IDV from Northern Ireland with cattle isolate D/bovine/Northern_Ireland/24280/2017 and noted the substitutions (Figure 2). We used amino acid numbering, as previously described, after removal of the first 16 N-terminal residues (*6*). We determined the degree of conservation at each substituted position by alignment of all 169 publicly available sequences (Appendix). 

**Figure 3:**
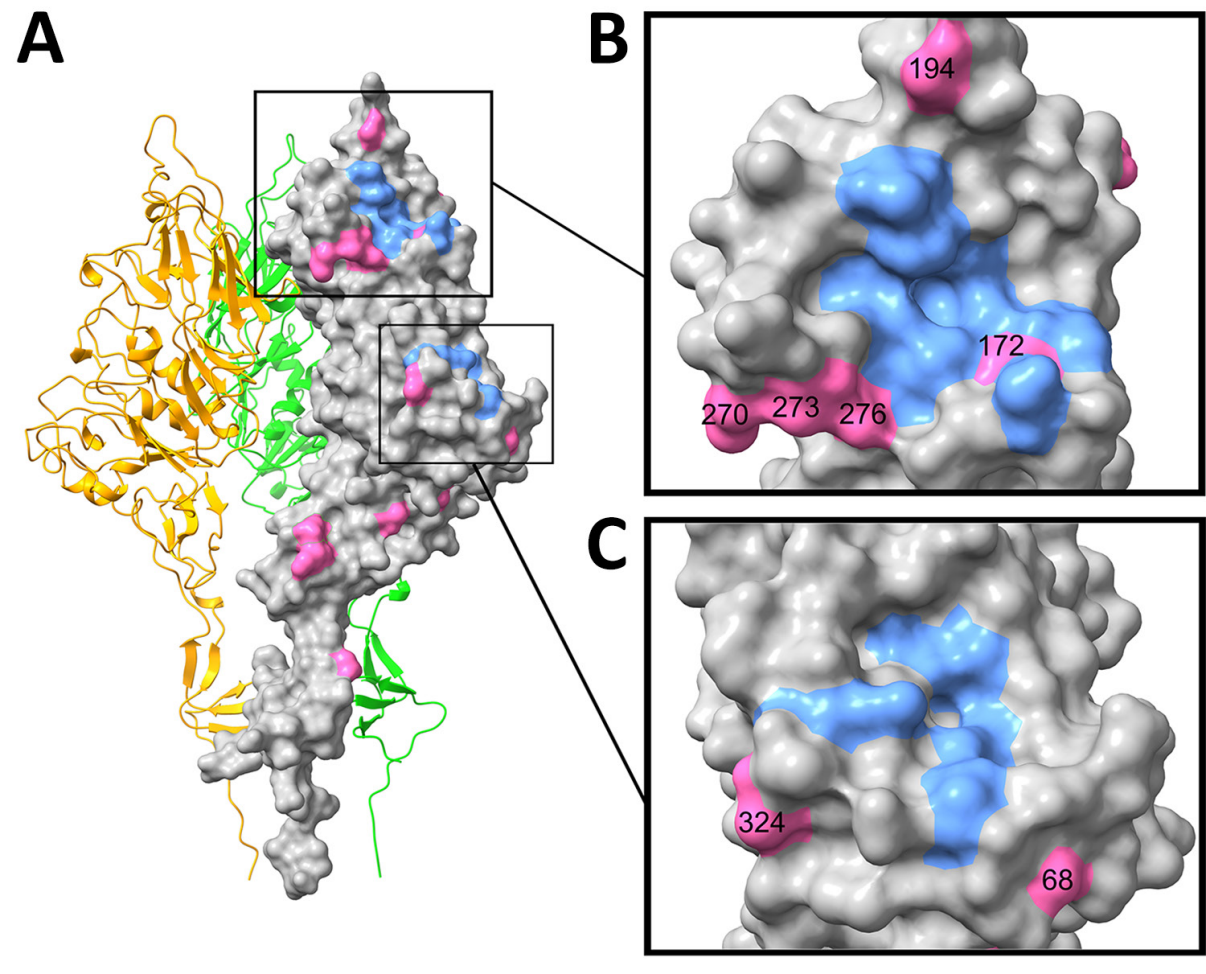
Locations of amino acid substitutions in swine-origin influenza D virus HEF protein structure, for high prevalence of influenza D virus infection in swine, Northern Ireland. A) Homology model of D/bovine/Northern_Ireland/24280/2017 hemagglutinin-esterase fusion protein generated with Swiss-Model (https://swissmodel.expasy.org) by using the structure of D/swine/Oklahoma/1334/2011 (https://doi.org/10.2210/pdb5e64/pdb) as a template. B) Close-up of receptor-binding site. C) Close-up of esterase. Homology model was annotated in ChimeraX version 1.8 (https://www.cgl.ucsf.edu/chimerax). Blue indicates receptor-binding site (F127, T171, A172, S173, W185, F229, Y231, T239, F240, V273, V275, F297) and esterase (S57, G85, N117, R326, D356, H359) residues. Pink indicates residues substituted in swine-origin isolates.

We mapped the location of the amino acid substitutions in the HEF protein structure onto a homology model of D/bovine/Northern_Ireland/24280/2017 HEF (Figure 3). Swine-origin strains differed from the bovine sequence at 5 positions around the receptor binding site. Of those positions, 3 were at highly conserved positions: G194R/K, I270M, and K276R. We only detected substitutions at 270 and 276 in samples from farm 2160, whereas we detected G194R/K in samples from 3 farms (2156, 2163, 2170). Swine-origin strains also differed from the bovine sequence at 2 positions around the esterase domain at the highly conserved positions A68V and A324S. We only detected substitutions at 68 in samples from farm 2163, whereas we detected A324S in samples from 3 farms (2160, 2168, 2179).

## Conclusions

Interspecies transmission from cattle, followed by some host adaptation and intraspecies spread, most likely initiates swine infection by IDV (*7*,*8*). Previous detection of IDV in swine have been associated with sample positivity rates mostly <1% (*2*). Combined with the low prevalence of IDV at the herd level, that positivity rate suggests intraspecies transmission is limited in swine. 

The organization of the agricultural sector in Northern Ireland may partly explain the high prevalence of IDV shown in our study. Multispecies farms containing both cattle and pigs are common and increase the likelihood of multiple IDV spillover events from cattle reservoirs. Indeed, phylogenetic analysis of swine-origin IDV in Northern Ireland identified several distinct genotypes, supporting the idea of repeated introductions from cattle (Appendix). However, the high rate of sample positivity observed on some farms is indicative of efficient pig-to-pig transmission. Furthermore, the continued detection of IDV on 1 farm (2170), 3 months after the initial detection, may represent continuous circulation of the virus within the herd. Those findings provide preliminary evidence for efficient adaptation of IDV to swine hosts and establishment within those populations. In addition, our data suggest that spread between farms might be a factor contributing to the high herd-level prevalence observed. The close genetic relatedness of some IDV strains isolated from both neighboring and geographically distant farms may indicate both local (direct) and long-distance (indirect) spread. Although common sources of indirect spread such as contaminated service vehicles should be considered, we must also consider cattle movements as a possible source of spread between swine units.

The molecular basis of IDV host adaptation has yet to be determined but likely includes changes that affect receptor binding. The receptor binding site in the IDV HEF protein is in a shallow cavity surrounded by secondary structure elements from the 170, 190, and 270 loops and the 230 helix (*6*). Substitutions in those secondary structure elements have the potential to modify receptor binding activity. For example, the open channel between the 230 helix and 270 loop is thought to enable broad cell tropism of IDV. Substitutions in those elements in the related influenza C virus HEF close that channel and restrict broad cell tropism (*6*). Candidate swine adaptations A236V and R268K have been previously identified after likely cattle-to-pig transmission (*7*), and L100F was observed after experimental infection of swine with a bovine-origin IDV isolate (*9*). In our study, we identified several additional candidates in both the receptor binding site and esterase. Of particular interest is mutation of G194 to a basic residue (R or K). Sequences from farms with the highest sample positivity rates (2163 and 2170) contained that mutation, and phylogenetic analysis indicated that strains from those farms were only distantly related and therefore seem to have independently converged on that adaptation. However, multiple routes to swine adaptation for IDV are likely, and alternative substitutions, such as the 270–273–276 triplet and the esterase changes at positions 68 and 324, should be examined further. Researchers should validate the proposed swine adaptive mutations, including receptor binding analysis and experimental transmission studies. Transmission routes of IDV to swine remain to be clarified but could be direct (e.g., contact with infected cattle) or indirect (e.g., contaminated vehicles).

Appendix Additional information for high prevalence of influenza D virus infection in swine, Northern Ireland.
