# Supplementary material for: High Prevalence of Influenza D Virus Infection in Swine, Northern Ireland
Source: Emerg Infect Dis. 2025 May;31(5):1023–7. doi: 10.3201/eid3105.241948 (PMC12044250; doi:10.3201/eid3105.241948)
Supplement: Appendix — Additional information for high prevalence of influenza D virus infection in swine, Northern Ireland. [file 24-1948-Techapp-s1.pdf]

Article DOI: https://doi.org/10.3201/eid3105.241948

EID cannot ensure accessibility for supplementary materials supplied by authors. Readers who have difficulty accessing supplementary content should contact the authors for assistance.

High Prevalence of Influenza D Virus Infection in Swine, Northern Ireland

Appendix

Supplemental Methods

Whole-genome sequences were determined directly from original clinical material for 2 positive samples per farm as described previously for swine influenza A virus (IAV) (*1*) by using modified primers specific for influenza D virus (IDV; primer sequences available upon request). Libraries were prepared from amplified DNA and sequenced with the DNA Prep kit on an Illumina MiSeq platform, and paired reads were aligned to the D/bovine/Northern_Ireland/24280/2017 reference genome by using the Burrows–Wheeler Aligner maximum exact matches (BWA-MEM) (v0.7.17-r1188) read aligner. Consensus sequences were deposited in GenBank (accession nos. PQ776796–PQ776889). Phylogenetic analysis showed that all 14 strains belonged to the D/OK lineage (Appendix Figures 1–8). Sequences from farms 2160, 2168, and 2179 were closely related and clustered together along with D/bovine/Northern_Ireland/24280/2017 across all 7 segments. Similarly, sequences from farms 2155 and 2163 clustered with D/bovine/Northern_Ireland/24280/2017 across 6 segments but had divergent NS segments. Sequences from farms 2156 and 2170 contained PB1, P3, and NP segments that clustered with D/bovine/Northern_Ireland/24280/2017 but had HEF, PB2, P42, and NS segments that clustered in separate clades that were only distantly related to the bovine sequences from Northern Ireland. This analysis confirmed that there are multiple IDV genotypes circulating in swine herds from Northern Ireland.

Swine-origin viruses from Northern Ireland differed from the bovine-origin sequence at 18 positions, with individual strains containing between 4 and 7 changes. K393R was the only change common to all swine-origin strains. However, position 393 is not conserved in IDV, with both K and R occurring at similar frequency. Swine strains had substitutions at 5 positions that are completely conserved in all other IDV sequences: E31K, A68V, T212K,

N220S, and D394N. In addition, substitutions at 9 highly conserved positions were noted: T40I, R147K, G194R/K, M260I, I270M, K276R, R292K, A324S, and S369N.

Reference

1. Lagan P, Hamil M, Cull S, Hanrahan A, Wregor RM, Lemon K. Swine influenza A virus infection dynamics and evolution in intensive pig production systems. Virus Evol. 2024;10:veae017. PubMed https://doi.org/10.1093/ve/veae017

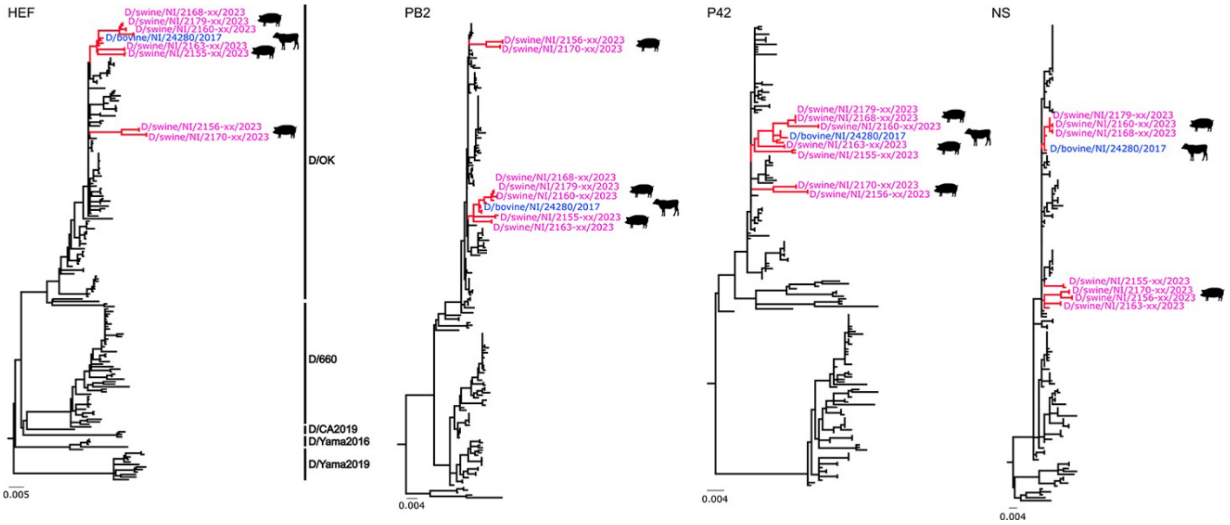

Appendix Figure 1. Maximum-likelihood influenza D virus phylogenetic trees displaying the relatedness of swine-origin influenza D virus HEF, PB2, P42, and NS sequences (pink text) to bovine-origin influenza D virus from Northern Ireland (blue text). Alignment of all publicly available influenza D virus sequences was performed by using the MAFFT v7 web server (https://mafft.cbrc.jp/alignment/server/). Alignments were viewed, trimmed to coding sequence, and curated using Aliview (https://ormbunkar.se/aliview/). Maximum likelihood phylogenetic trees were obtained for each segment by using the IQTREE web server (http://iqtree.cibiv.univie.ac.at/), performing ultrafast bootstrap resampling analysis with 1,000 iterations and by using the best-fitted nucleotide substitution model selected by ModelFinder. Trees were visualized, rooted at midpoint, and annotated by using FigTree v1.4.4 (http://tree.bio.ed.ac.uk/software/figtree/). Scale bar indicates number of nucleotide substitutions per site.

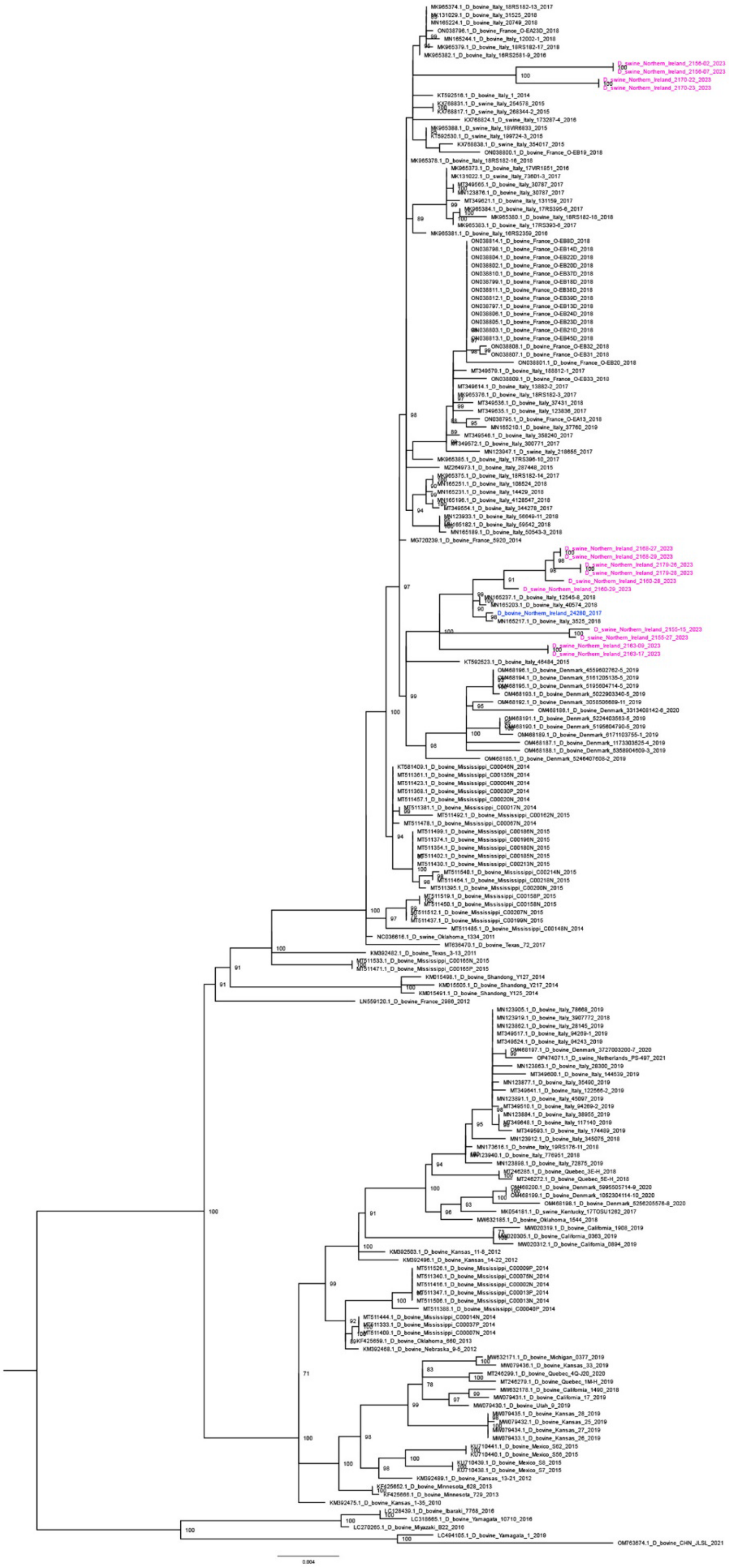

Appendix Figure 2. Maximum-likelihood influenza D virus phylogenetic tree displaying the relatedness of swine-origin influenza D virus PB2 coding sequences (pink text) to bovine-origin influenza D virus from Northern Ireland (blue text). Scale bar indicates substitutions per site.

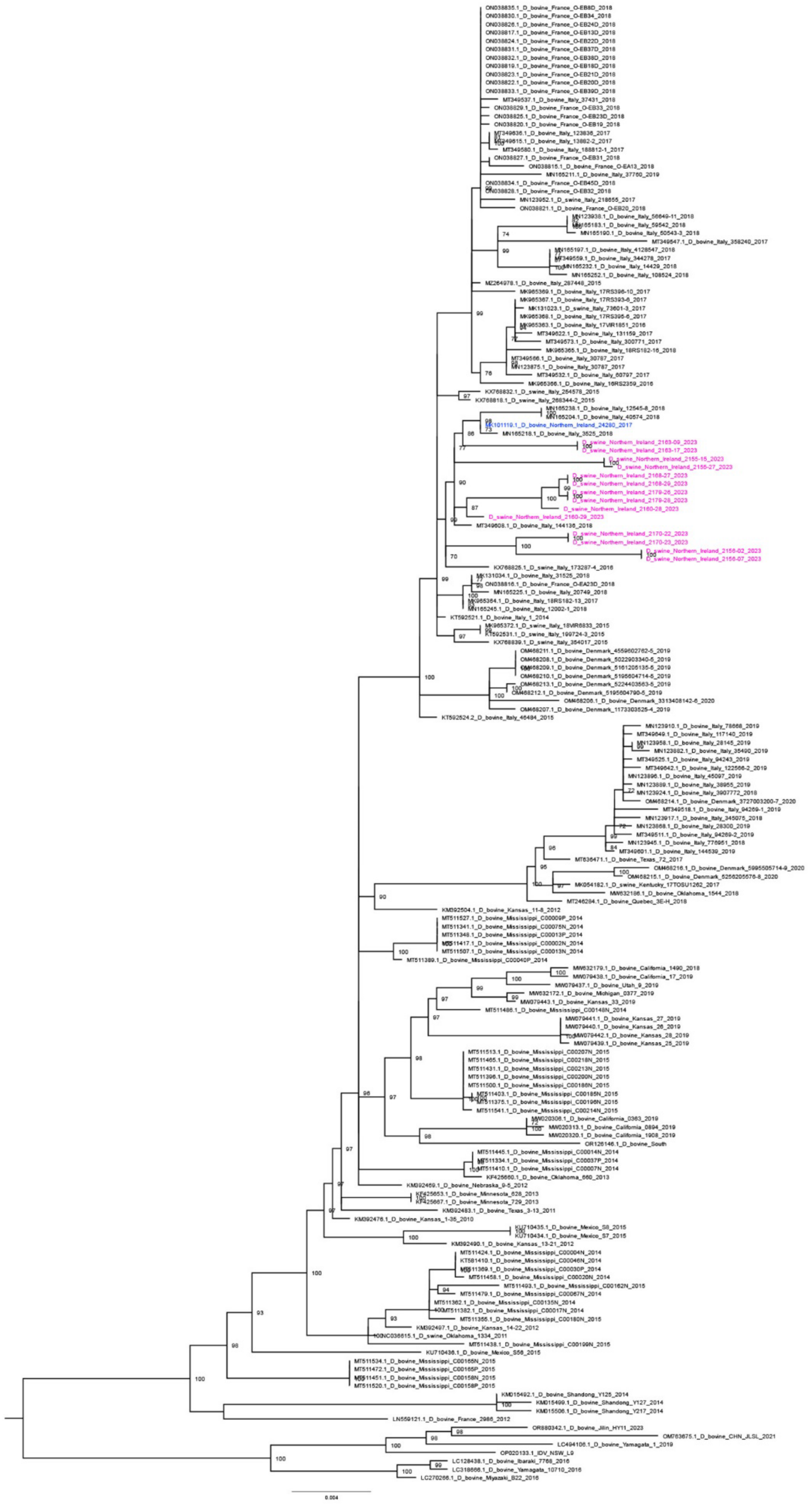

Appendix Figure 3. Maximum-likelihood influenza D virus phylogenetic tree displaying the relatedness of swine-origin influenza D virus PB1 coding sequences (pink text) to bovine-origin influenza D virus from Northern Ireland (blue text). S Scale bar indicates substitutions per site.

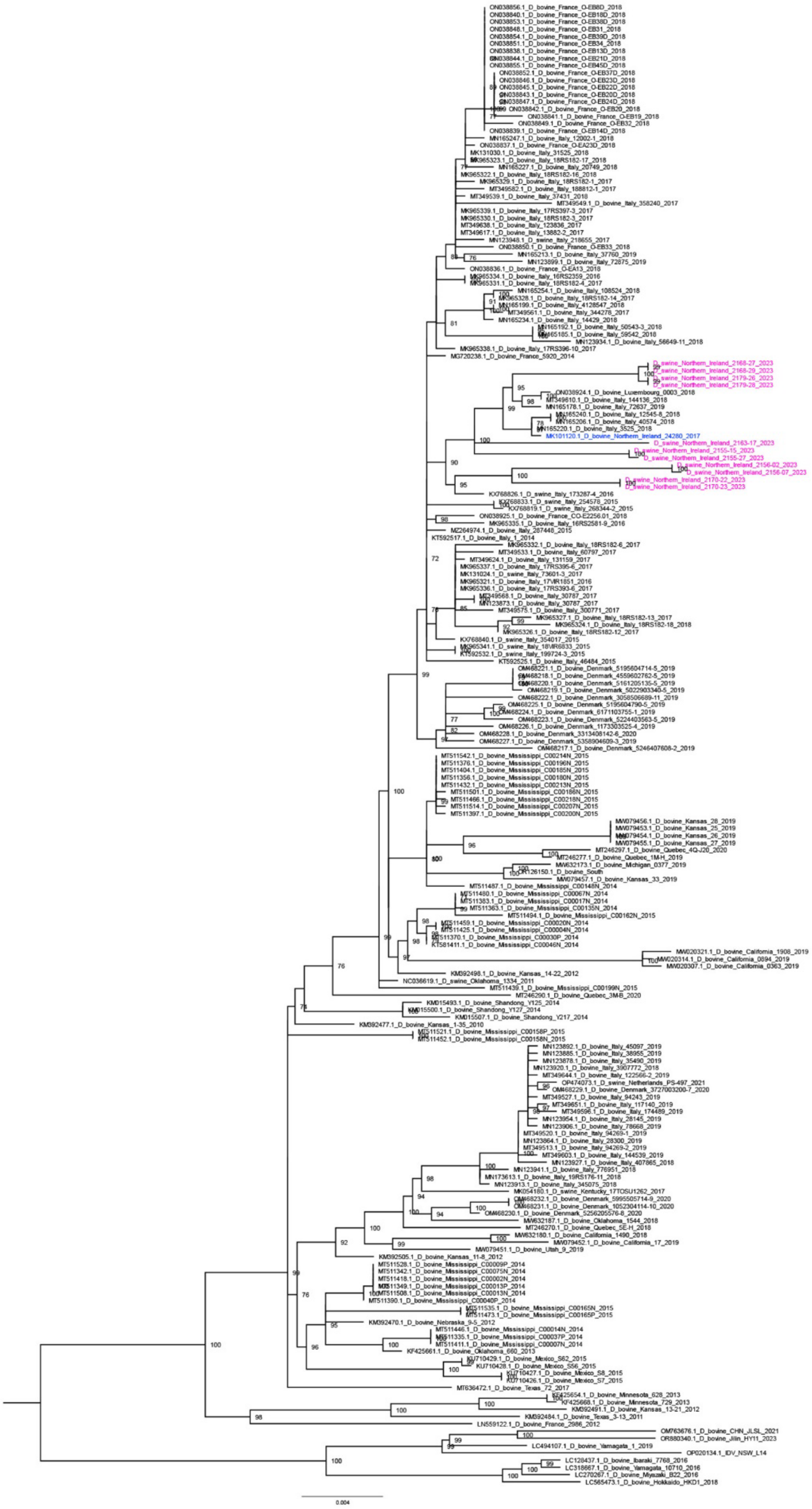

Appendix Figure 4. Maximum-likelihood influenza D virus phylogenetic tree displaying the relatedness of swine-origin influenza D virus P3 coding sequences (pink text) to bovine-origin influenza D virus from Northern Ireland (blue text). Scale bar indicates substitutions per site.

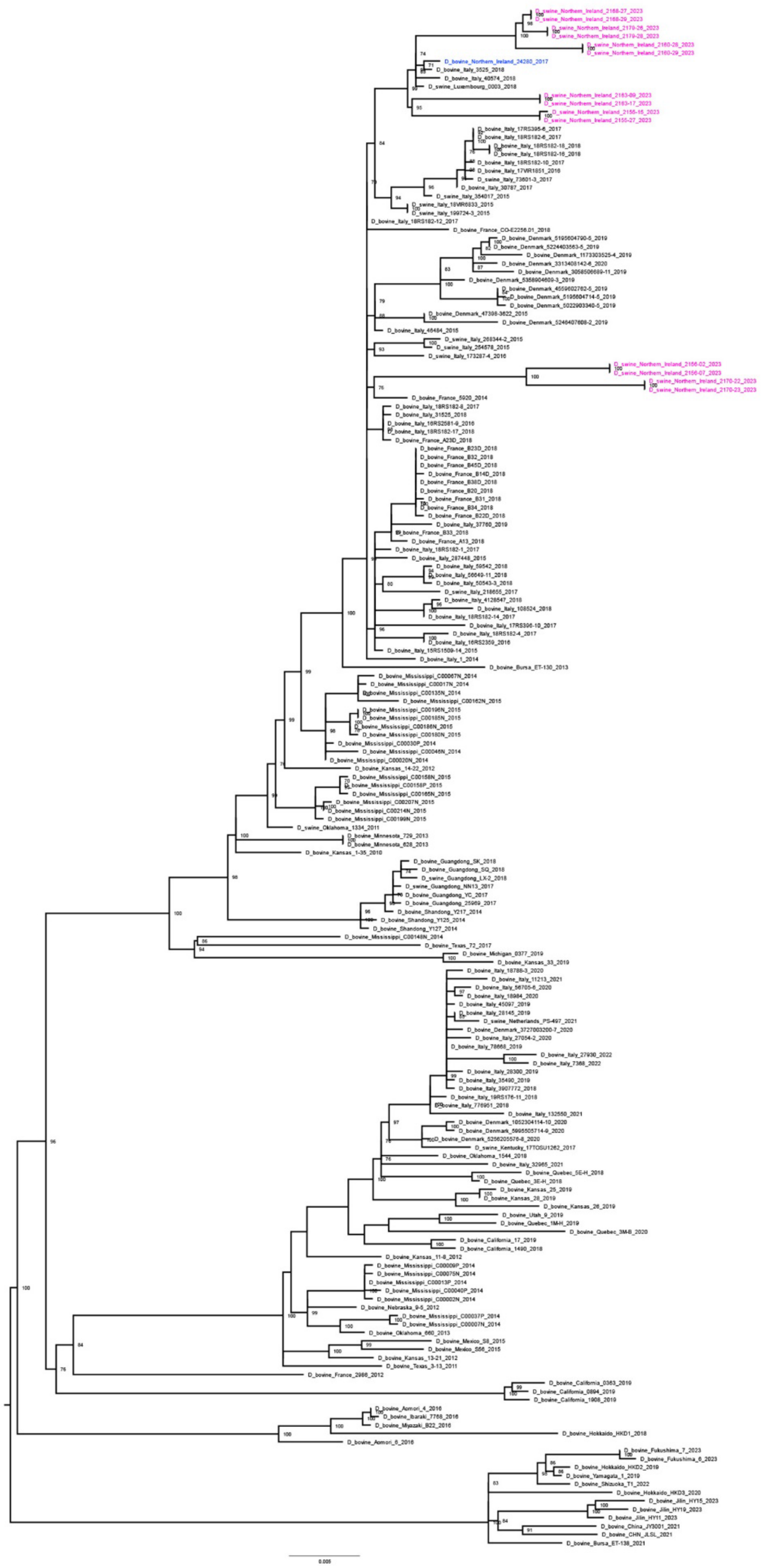

Appendix Figure 5. Maximum-likelihood influenza D virus phylogenetic tree displaying the relatedness of swine-origin influenza D virus HEF coding sequences (pink text) to bovine-origin influenza D virus from Northern Ireland (blue text). Scale bar indicates substitutions per site.

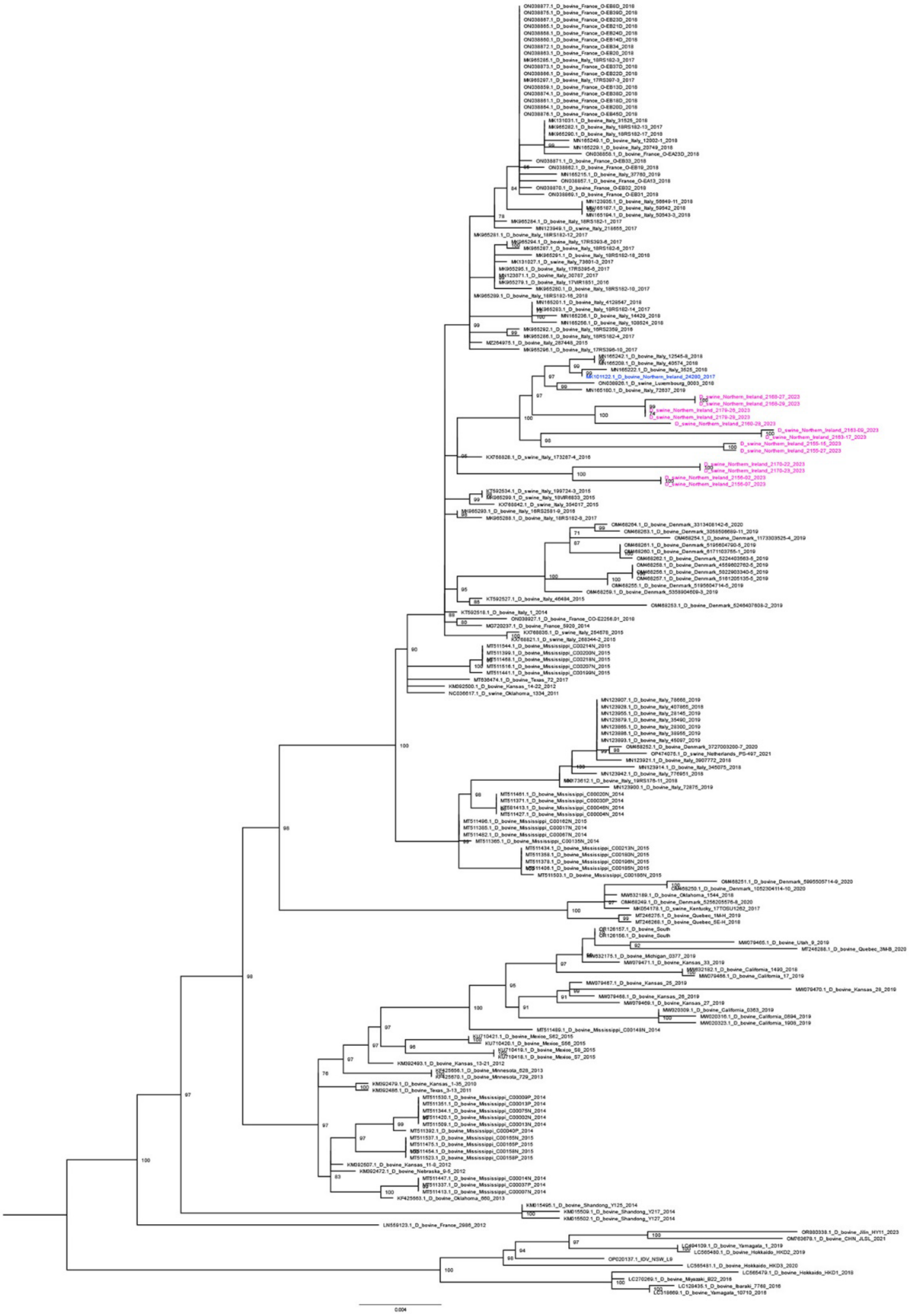

Appendix Figure 6. Maximum-likelihood influenza D virus phylogenetic tree displaying the relatedness of swine-origin influenza D virus NP coding sequences (pink text) to bovine-origin influenza D virus from Northern Ireland (blue text). Scale bar indicates substitutions per site.

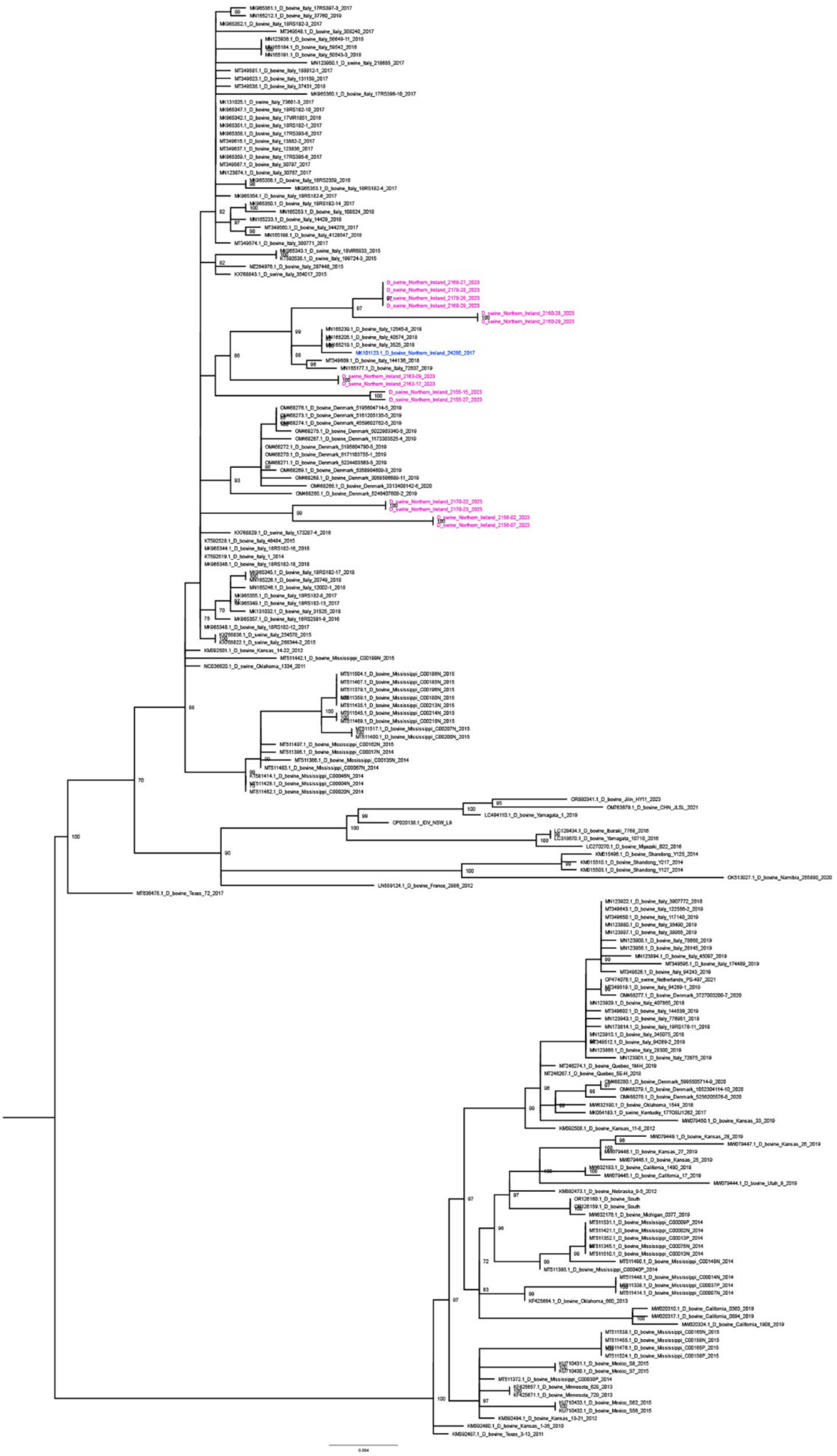

Appendix Figure 7. Maximum-likelihood influenza D virus phylogenetic tree displaying the relatedness of swine-origin influenza D virus P42 coding sequences (pink text) to bovine-origin influenza D virus from Northern Ireland (blue text). Scale bar indicates substitutions per site.

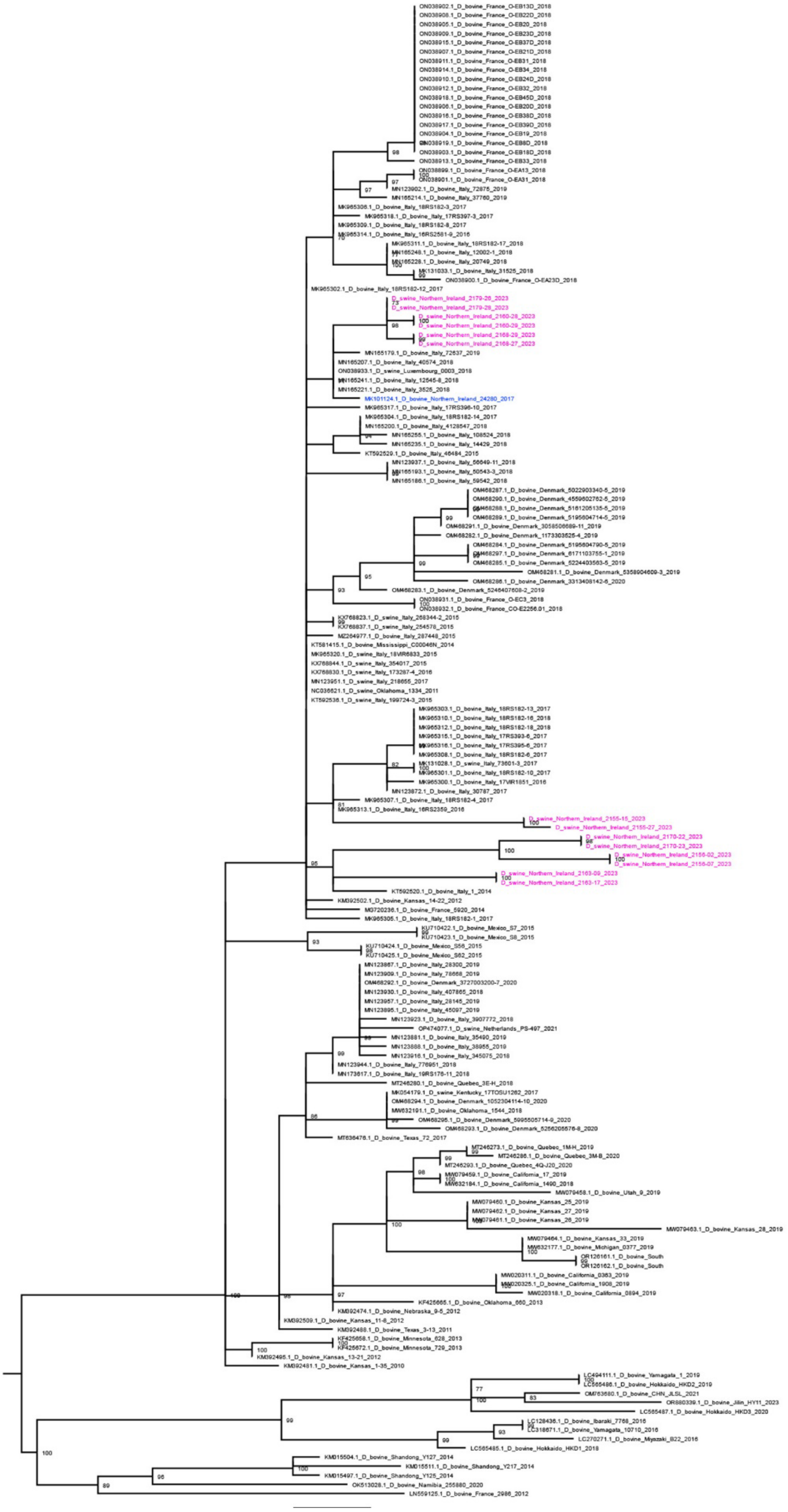

Appendix Figure 8. Maximum-likelihood influenza D virus phylogenetic tree displaying the relatedness of swine-origin influenza D virus NS coding sequences (pink text) to bovine-origin influenza D virus from Northern Ireland (blue text). Scale bar indicates substitutions per site.
